# Microvascular alterations in patients with SARS-COV-2 severe pneumonia

**DOI:** 10.1186/s13613-020-00680-w

**Published:** 2020-05-20

**Authors:** Elisa Damiani, Andrea Carsetti, Erika Casarotta, Claudia Scorcella, Roberta Domizi, Erica Adrario, Abele Donati

**Affiliations:** Anesthesia and Intensive Care Unit, Department of Biomedical Sciences and Public Health, Università Politecnica delle Marche, via Tronto 10/a, 60126 Ancona, Italy

To the Editor,

Multiple evidences suggest that pulmonary microcirculatory dysfunction may play a key role in the pathogenesis of SARS-COV-2 severe pneumonia.

SARS-COV-2 uses the angiotensin converting enzyme 2 (ACE2) as its receptor [1]. ACE2 normally functions as a negative regulator of the renin–angiotensin system (RAS) [1]. RAS dysregulation leads to increased vascular permeability, inflammation and pneumocyte apoptosis [1]. Pulmonary microvascular leakage may result in lung oedema and impaired lung function.

Severe Coronavirus disease 2019 (COVID-19) is frequently complicated by coagulopathy and markedly elevated D-dimer is associated with poor prognosis [2]. The formation of micro-thrombi in the lung vessels likely contributes to ventilation/perfusion mismatch and impairs gas exchange.

In this study, we reviewed data from mechanically ventilated patients with SARS-COV-2 severe pneumonia admitted to an intensive care unit (ICU) of Ancona (Italy) in March 2020, who underwent an evaluation of the sublingual microcirculation by means of incident dark field videomicroscopy (Cytocam, Braedius Medical, Amsterdam, NL). The protocol of this retrospective observational study was approved by the local Ethics Committee (Comitato Etico Regionale delle Marche).

The Cytocam is a third generation handheld video-microscope that enables the non-invasive, real-time, in vivo visualization of the microcirculation [3]. This technique is routinely applied in our ICU to monitor microvascular perfusion. Three videos from different sublingual areas were recorded with adequate contrast and focus and without pressure artefacts. The videos were analysed offline with dedicated software (Automated Vascular Analysis 3.2, Microvision Medical, Amsterdam, NL) to obtain parameters of vessel density (total vessel density [TVD], perfused vessel density [PVD]) and blood flow quality (microvascular flow index [MFI], percentage of perfused vessels [PPV] and flow heterogeneity index [HI]), as described elsewhere [3].

Data are presented as mean (± standard deviation) or median [1st–3rd quartile], based on the distribution of the variable of interest. The Spearman’s rho was calculated to evaluate correlations between variables with a significance level of 0.05 (IBM SPSS Statistics for Windows, Version 21.0. Armonk, NY: IBM Corp).

Among 29 patients with SARS-COV-2 severe pneumonia who were admitted to our ICU during the study period, 12 patients underwent microcirculatory evaluation. Patients’ characteristics are reported in Table 1. Microvascular parameters for vessels smaller than 20 μm were: TVD 15.3 [14.5–17.1] mm/mm^2^; PVD 14.9 [14.1–16.9] mm/mm^2^; PPV 97.3 [95.1–98.8] %; MFI 2.9 [2.6–3]; HI 0.3 [0–0.4]. D-Dimer levels were inversely correlated with PVD (Spearman rho = − 0.70, *p* = 0.016) and TVD (rho = − 0.645, *p* = 0.032) (Fig. 1). D-Dimer levels were also inversely correlated with PaO_2_/FiO_2_ (rho = − 0.609, *p* = 0.047). PVD tended to decrease with increasing driving pressure values (rho = − 0.691, *p* = 0.086).

**Table 1:** Patients’ characteristics

Male (n, %)	10 (83.3%)
Age (years)	56 (10)
BMI (kg/m^2^)	31.6 (5.4)
Comorbidities (*n*, %)	
Dyslipidemia	4 (33.3%)
Hypertension	3 (25.0%)
Diabetes type 2	2 (16.7%)
Ischemic cardiomyopathy	2 (16.7%)
Tidal volume (ml)	421 (190)
RR (breath/min)	13 (3)
Pplat (cmH_2_O)	27 (5)
PEEP (cmH_2_O)	10 (8.6; 13.8)
∆P (cmH_2_O)	13 (4)
Cstat (ml/cmH_2_O)	52 (37)
FiO_2_	0.40 (0.35; 0.48)
PaO_2_/FiO_2_ (mmHg)	207 (88)
VV-ECMO (n, %)	6 (50.0%)
CRRT (n, %)	2 (16.7%)
MAP (mmHg)	88 (13)
HR (beat/min)	86 (23)
Lactate (mmo/l)	1.16 (0.41)
WBC (×10^9^/l)	14.12 (5.13)
IL-6 (pg/ml)	138 (18.5; 338)
D-Dimer (ng/ml)	788 (717; 5536)
Noradrenaline	
*n* (%)	9 (75%)
mcg/kg/min	0.24 (0.14)
Propofol	
*n* (%)	9 (75%)
mg/kg/h	2.5 (0.46)
Midazolam	
*n* (%)	9 (75%)
mg/kg/h	0.26 (0.12)
Remifentanil	
*n* (%)	12 (100%)
mcg/kg/min	0.1 (0.85; 0.1)

Data reported as *n*. (%); mean (standard deviation); median (interquartile range)

*BMI* body mass index, *CRRT* continuous renal replacement therapy, *Cstat* static compliance of respiratory system, *FiO*_*2*_ inspiratory fraction of oxygen, *HR* heart rate, *IL-6* interleukin 6, *MAP* mean arterial pressure, *PaO*_*2*_ arterial partial pressure of oxygen, *∆P* driving pressure, *PEEP* positive end expiratory pressure, *Pplat* plateau pressure, *RR* respiratory rate, *VV-ECMO* veno-venous extracorporeal membrane oxygenation, *WBC* white blood cells

**Fig. 1:**
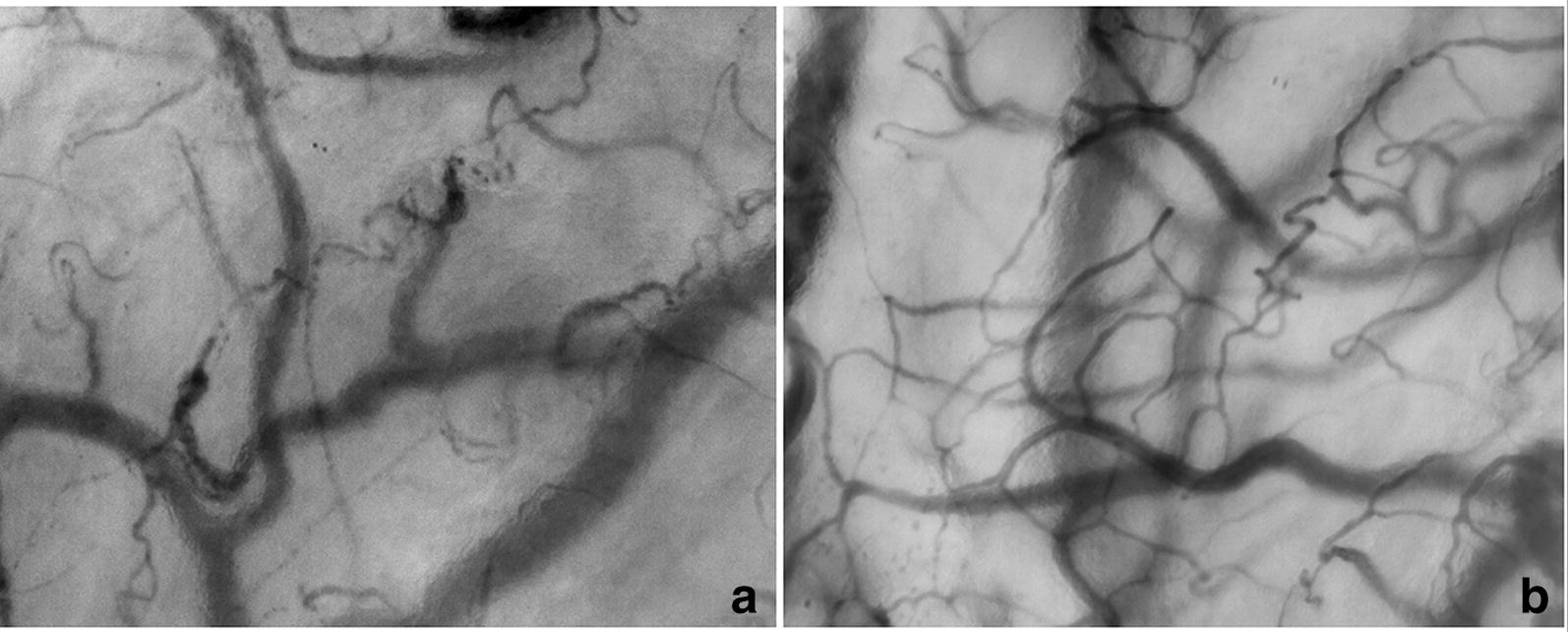
Sublingual microcirculation of two patients with SARS-COV2 severe pneumonia. Patient (**a**) showed a significantly lower perfused vessel density as compared to patient (**b**). D-dimer levels were 6367 ng/ml in patient (**a**) and 741 ng/ml in patient (**b**). PaO_2_/FiO_2_ ratio was 74 mmHg in patient (**a**) and 247 mmHg in patient (**b**)

This is the first study that evaluated microcirculatory blood flow in COVID-19 patients. Microvascular alterations are associated with mortality in critically ill patients [3]. In a general population of 97 critically ill patients, we previously reported a PVD of 19.3 ± 4.4 mm/mm^2^ [3], which seems significantly higher in comparison with the value observed in this sample of COVID-19 patients. Varga et al. recently reported signs of endotheliitis in several organs in patients with SARS-COV-2 infection, suggesting systemic microvascular dysfunction that may account for tissue hypoperfusion, inflammation and a procoagulant state [4].

Sublingual microcirculatory blood flow was significantly compromised in patients with severe influenza A (H1N1) infection [5]. In acute respiratory distress syndrome, increased heterogeneity of sublingual microvascular perfusion was related to an increase in dead-space ventilation, suggesting a role of microcirculatory dysfunction in ventilation/perfusion mismatching [6].

Our report supports a link between coagulopathy and microvascular perfusion disturbances in patients with SARS-COV-2 severe pneumonia. Further studies are needed to demonstrate a cause–effect relationship, clarify the role of microcirculatory disturbances on lung function and indicate potential implications for therapy.

## Data Availability

All data generated or analysed during this study are included in this published article.

## Abbreviations

SARS-COV-2: Severe Acute Respiratory Syndrome Coronavirus 2
ACE2: Angiotensin converting enzyme 2
RAS: Renin–angiotensin system
COVID-19: Severe Coronavirus disease 2019
ICU: Intensive care unit
TVD: Total vessel density
PVD: Perfused vessel density
MFI: Microvascular flow index
PPV: Percentage of perfused vessels
HI: Flow heterogeneity index
